# RETRACTED: Kang et al. Predicting Safe Liver Resection Volume for Major Hepatectomy Using Artificial Intelligence. *J. Clin. Med.* 2024, *13*, 381

**DOI:** 10.3390/jcm15218590

**Published:** 2026-11-04

**Authors:** Chol Min Kang, Hyung June Ku, Hyung Hwan Moon, Seong-Eun Kim, Ji Hoon Jo, Young Il Choi, Dong Hoon Shin

**Affiliations:** 1 Department of Applied Biomedical Engineering, The Johns Hopkins University, Baltimore, MD 21287, USA; kcholmin@gmail.com; 2 Chang Kee-Ryo Memorial Liver Institute, Kosin University College of Medicine, Busan 49267, Republic of Korea; hjkoo1995@gmail.com (H.J.K.); 21cjjh@daum.net (J.H.J.); tsojc7@gmail.com (Y.I.C.); surgeonshin@naver.com (D.H.S.); 3 Division of Hepatobiliary-Pancreas and Transplantation, Department of Surgery, Kosin University Gospel Hospital, Busan 49267, Republic of Korea; 4 Department of Applied Artificial Intelligence, Seoul National University of Science and Technology, Seoul 01811, Republic of Korea; sekim@seoultech.ac.kr

The journal retracts the article titled “Predicting Safe Liver Resection Volume for Major Hepatectomy Using Artificial Intelligence” [1], cited above.

Following publication, concerns were brought to the attention of the Editorial Office regarding the accuracy of cited sources and the reliability of the findings presented in the paper [1].

In accordance with the journal’s standard procedures, the Editorial Office and Editorial Board conducted an investigation that confirmed the undisclosed use of artificial intelligence and the presence of a significant number of inaccurate and non-existent references. During the investigation, additional concerns were identified, including errors in the calculation of the ALBI score affecting Figures 6 and 7 and inaccuracies in the data presented in Table 1. Although the authors cooperated with the investigation, the Editorial Board determined that the explanations and supporting documentation provided were insufficient to resolve the concerns identified, and the required changes were deemed too extensive to be addressed through a correction. Consequently, the Editorial Board lost confidence in the reliability of the findings and decided to retract this publication [1], in accordance with MDPI’s retraction policy.

This retraction was approved by the Editor-in-Chief of the *Journal of Clinical Medicine*.

The authors have been informed of this retraction and have indicated their agreement with this decision.
